# Direct Oral Anticoagulants in Special Patient Populations

**DOI:** 10.3390/jcm13010216

**Published:** 2023-12-29

**Authors:** Asa Kessler, Yotam Kolben, Gal Puris, Martin Ellis, Mordechai Alperin, Vered Simovich, Hila Lerman Shivek, Mordechai Muszkat, Yoram Maaravi, Yitschak Biton

**Affiliations:** 1 Heart Institute, Hadassah Medical Center and Faculty of Medicine, Hebrew University of Jerusalem, Jerusalem 9112002, Israel; asak@hadassah.org.il (A.K.); yotamkol@hadassah.org.il (Y.K.); 2 Faculty of Medicine, Institute for Research in Military Medicine, Hebrew University of Jerusalem, Israel Defense Force Medical Corps, Jerusalem 9112002, Israel; gal.puris@mail.huji.ac.il; 3 Hematology Institute and Blood Bank, Meir Medical Center, Kfar Saba 4428164, Israel; martinel@clalit.org.il; 4 Faculty of Medicine, Tel Aviv University, Tel Aviv 6997801, Israel; 5 Department of Family Medicine, The Ruth & Bruce Rappaport Faculty of Medicine, Technion-Israel Institute of Technology, Haifa 3200003, Israel; mordechaial@clalit.org.il; 6 Clalit Health Services, Haifa and Western Galilee District, Tel Aviv 6209804, Israel; 7 Maccabi Health Care Services, Tel Aviv 6209804, Israel; simovich_v@mac.org.il; 8 Hospital Pharmacy Department, Hospitals Division, Clalit Health Services, Tel Aviv 6209804, Israel; hilalerm@clalit.org.il; 9 Institute for Drug Research, School of Pharmacy, Faculty of Medicine, Hebrew University of Jerusalem, Jerusalem 9112002, Israel; 10 Department of Medicine, Hadassah Medical Center, Faculty of Medicine, Hebrew University of Jerusalem, Mt. Scopus, Jerusalem 9112002, Israel; muszkatm@hadassah.org.il; 11 The Jerusalem Institute of Aging Research, Faculty of Medicine, Hebrew University of Jerusalem, Jerusalem 9112002, Israel; maaravi@hadassah.org.il; 12 Department of Geriatrics and Rehabilitation and the Center for Palliative Care, Hadassah Medical Center, Jerusalem 9371125, Israel

**Keywords:** direct oral anticoagulants, atrial fibrillation, special populations

## Abstract

Anticoagulants are a cornerstone of treatment in atrial fibrillation. Nowadays, direct oral anticoagulants (DOACs) are extensively used for this condition in developed countries. However, DOAC treatment may be inappropriate in certain patient populations, such as: patients with chronic kidney disease in whom DOAC concentrations may be dangerously elevated; frail elderly patients with an increased risk of falls; patients with significant drug–drug interactions (DDI) affecting either DOAC concentration or effect; patients at the extremes of body mass in whom an “abnormal” volume of distribution may result in inappropriate drug concentrations; patients with recurrent stroke reflecting an unusually high thromboembolic tendency; and, lastly, patients who experience major hemorrhage on an anticoagulant and in whom continued anticoagulation is deemed necessary. Herein we provide a fictional case-based approach to review the recommendations for the use of DOACs in these special patient populations.

## 1. Introduction

Atrial fibrillation (AF) and atrial flutter are the most commonly diagnosed cardiac arrythmias worldwide [1]. AF poses a great burden on society as it is a major risk factor for the development of stroke, heart failure and systemic embolism [2]. Forty to sixty million individuals worldwide have AF, about twice-fold the prevalence in 1990 [3,4]. AF is associated with increased mortality and morbidity and strategies focused on screening for AF and prevention of complications have been proposed [5].

**Figure 1:**
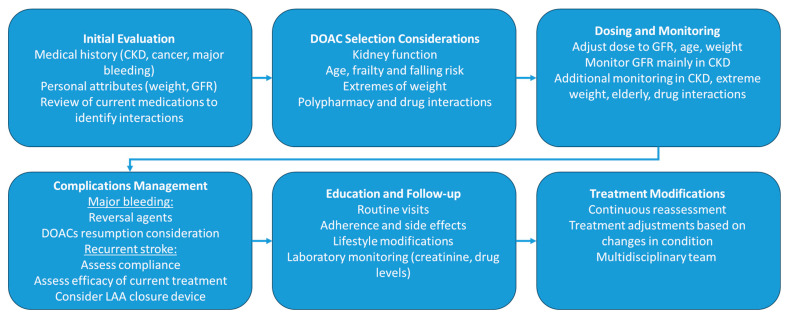
Algorithm for the initiation and management of DOACs in special populations. CKD, chronic kidney disease; GFR, glomerular filtration rate.

AF is associated with a five-fold increase in the risk of stroke [6], for an annual rate of ~2%, while atrial flutter is associated with a lower risk [7]. Stroke prevention is therefore a cornerstone of treatment in these patients. Consequently, guidelines recommend anticoagulation in patients at high risk of stroke or systemic embolization, as calculated by the CHA_2_DS_2_-VASc (congestive heart failure, hypertension, age, diabetes mellitus, prior stroke or transient ischemic attack, vascular disease and sex category) score. Direct oral anticoagulants (DOACs) are recommended in all patients with AF without a mechanical heart valve or with moderate to severe mitral stenosis [5,8,9]. Despite these recommendations, data regarding the use of DOACs in specific populations are lacking. Pivotal studies for the use of DOACs have systematically excluded patients with severe renal impairment, extremes of body weight and higher ischemic and bleeding risks, leading to uncertainty regarding the efficacy and safety of DOACs in these patients. The leading paradigms focus on patient-centered care with a holistic view. The ABC pathway used in the European guidelines is based on identifying each patients’ risks, symptoms, and comorbidities and offering a management strategy based on integrated care [10]. This paper will focus on current data and considerations regarding the use of DOACs in special populations (Figure 1) utilizing fictional scenarios that emphasize the importance of tailored considerations for optimal management while acknowledging the lack of evidence-based best-practice recommendations for these patients. The patient cases described in this publication are fictional and do not represent events, or a response, from actual patients. The authors developed this fictional case for educational purposes only.

## 2. DOACs in Chronic Kidney Disease

*A 67-year-old female presents with new-onset AF. She has heart failure with preserved ejection fraction, hypertension, diabetes mellitus, dyslipidemia and chronic renal failure with a baseline creatinine level of 1.7–2.3 mg/dL. Her medications include an angiotensin converting enzyme inhibitor, a calcium channel blocker, a statin, a proton pump inhibitor, aspirin, and insulin. Is it appropriate to use DOACs? Compared to VKA, what are the levels of safety and efficacy, taking into consideration a dose adjustment?*

Renal dysfunction poses great challenges for stroke prevention in patients with AF because of the partial renal elimination of all DOACs which is 80% for dabigatran, 50% for edoxaban, 35% for rivaroxaban and 27% for apixaban [11]. General considerations for anticoagulation include the degree of renal impairment according to estimated glomerular filtration rates and whether dialysis is needed. Different dose-reduction recommendations for these drugs exist, and what further affects the pharmacologic attributes of DOACs is the presence of kidney disease.

The approved standard dose for apixaban is 5 mg BID (twice daily) for the prevention of stroke in non-valvular AF with a reduction to 2.5 mg BID recommended if at least two of the following features are present: age ≥ 80, weight < 60 kg or serum creatinine ≥ 1.5 mg/dL; meanwhile, some recommend standard dosing in Creatinine clearance (CrCl) > 30 mL/min and dose reduction in CrCl 15 to 30 mL/min [12,13]. For dabigatran, the standard dose is 150 mg BID, with dose reduction to 110 mg BID for patients with higher bleeding risk. In the United States, dabigatran 75 mg BID for CrCl 15–29 mL/min is approved. The edoxaban standard dose is 60 mg QD (once daily) with dose reduction recommended in weight < 60 kg, CrCl 15–49 mL/min or concomitant use of a strong p-glycoprotein inhibitor. The rivaroxaban standard dose is 20 mg QD with reduction to 15 mg QD if CrCl is 15–49 mL/min [13] (Table 1). Of note, no DOAC is approved below a CrCl of 15 mL/min in Israel and Europe.

The four pivotal randomized controlled trials (RCTs) comparing the use of DOACs to warfarin in patients with AF have excluded patients with severe renal impairment. In RE-LY, comparing dabigatran to warfarin, ROCKET-AF, comparing rivaroxaban to warfarin and ENGAGE AF TIMI 48, comparing edoxaban to warfarin, a cutoff of creatinine clearance of less than 30 mL/min was set for exclusion [14,15,16]. In contrast, in ARISTOTLE, comparing apixaban to warfarin, a lower cutoff of 25 mL/min was used [17]. These exclusion criteria present a significant management problem considering the high prevalence of AF in advanced kidney disease, reaching 15–20% in patients receiving hemodialysis, and considering the higher risk of stroke and thromboembolism in this sub-group [18,19].

Despite the evidence gap that exists regarding the use of DOACs in renal dysfunction, a large meta-analysis showed that DOACs were associated with similar or better outcomes than warfarin in patients with varying degrees of pre-dialysis renal impairment, suggesting that DOACs may be a reasonable choice for stroke prevention in AF regardless of the extent of kidney dysfunction [20].

Data regarding the use of DOACs in hemodialysis are limited with only a few RCTs focused on the safety and efficacy of apixaban in end-stage renal disease, this despite the higher risk of stroke and thromboembolism in this population, regardless of the CHA_2_DS_2_-VASc score [18]. The RENAL-AF trial, comparing apixaban to warfarin, was stopped prematurely because of enrollment challenges after including only 154 patients. Although there were numerically more bleeding events and deaths in the apixaban group, no conclusion regarding major or clinically relevant nonmajor bleeding can be made in this underpowered trial [21]. The AXADIA-AFNET 8 trial, comparing apixaban to phenprocoumon, showed no statistically significant difference in safety or efficacy outcomes, although it was underpowered due to the failure to reach the calculated number of patients and events [22] (Table 2). Data from the ongoing SAFE-D trial, which recruited 151 patients, have yet to be published [23].

Two large retrospective studies published including nearly 100,000 patients aimed to evaluate the safety and efficacy of DOACs compared to warfarin in patients with renal failure [24,25]. Apixaban was associated with a lower risk of major bleeding compared to warfarin in one (HR 0.72, 95% CI, 0.59–0.87) [21], and a higher bleeding risk in warfarin, dabigatran and rivaroxaban compared to apixaban in the other (HR, 95% CI 1.4 1.07–1.88, HR 2.07, 95% CI 1.42–3.01, HR 1.92, 95% CI 1.25–2.94, respectively) [24,25]. In addition, the standard dose of apixaban 5 mg BID compared to warfarin and to apixaban 2.5 mg BID was associated with a reduction in thromboembolic (HR 0.64, 95% CI 0.42–0.97 and HR 0.61, 95% CI 0.37–0.98, respectively) and mortality risk (HR 0.63, 95% CI 0.46–0.85 and HR 0.64, 95% CI 0.45–0.92, respectively) [24,25].

The 2019 American Heart Association/American College of Cardiology/Heart Rhythm Association (AHA/ACC/HRS) focused update of the 2014 guideline for the management of patients with AF recommends that in patients with severe renal impairment with creatinine clearance lower than 15 mL/min or on hemodialysis, apixaban may be considered [9], whereas the European Heart Rhythm Association is somewhat more conservative stating that the lack of strong evidence for the use of DOACs in this patient population should be communicated to the patients and warrants a high degree of individualization [13].

*This patient’s chronic kidney disease should not prevent her from receiving a DOAC as her estimated CrCl is greater than 15 mL/min.Based on her renal function being in a steady state, she should receive a reduced dose for the chosen DOAC. In the event of further renal deterioration, hard evidence regarding anticoagulation in patients with end-stage renal disease and patients on dialysis is absent. Thus, according to the European [5] and American (class 2a recommendation) [26] guidelines, the decision whether to use a DOAC or vitamin K antagonists (VKAs) should be made on a patient-centered basis. However, data from clinical trials to support the use of DOACs while on dialysis is accumulating.*

## 3. DOACs in Advanced Age

*A 91-year-old lucid woman who uses a walker presents to the clinic because a routine pacemaker examination demonstrated many episodes of AF on her electrocardiograph tracing. Her medical history is positive for smoking-related chronic lung disease, diabetes with peripheral neuropathy, osteoporosis and heart failure with preserved ejection fraction. She underwent a transcatheter aortic valve implantation one year ago, and pacemaker implantation due to complete atrio-ventricular block after the procedure. She weighs 45 kg, and her laboratory results show creatinine 1.3 mg/dL, hemoglobin 13 g%, platelets 180 × 10^3^/μL, and her INR is 1.1. Her medications include clopidogrel, bisoprolol, simvastatin, furosemide, insulin and empagliflozin. She is asymptomatic. When taking into account the advanced age with all its accompanying conditions, does the use of DOACs compared to VKAs demonstrate superior safety and efficacy?*

The risk of AF increases with age. According to the European Society of Cardiology (ESC) guidelines, screening for AF is probably cost-effective in older adults (age > 65 years) [5]. Elderly patients may pose challenges in managing oral anticoagulants due to frailty, falls, dementia, multiple co-morbidities and polypharmacy, which increase the risk of bleeding, thrombotic events, and mortality [27]. Furthermore, there is an underrepresentation of elderly patients in RCTs and consequently guidelines lack recommendations for these patients [28]. Because of these challenges, there is potentially inappropriate underuse of anticoagulants among the elderly [29].

An analysis performed in the pre-DOAC era showed that an elderly patient has to fall 295 times in one year to justify the lack of anticoagulation treatment [30] given the potential clinical benefit regarding the stroke vs. bleeding risk associated with falls. Despite this and possibly because of overestimation of fall-related bleeding in the elderly, only 40–60% of suitable candidates receive anticoagulation [27].

The introduction of DOACs has improved the benefit–risk ratio of anticoagulation with several RCTs demonstrating the superiority of DOACs over VKAs in AF, even in older adults (Table 3). A subgroup analysis of the ARISTOTLE trial showed that the benefits of apixaban in preventing stroke were present with lower bleeding rates compared to warfarin and were consistent among all age groups, including the elderly population (age > 80 years) [31]. Similarly, a subgroup analysis of patients >85 years in the AVERROES trial of apixaban versus aspirin in preventing stroke in patients with AF who could not take warfarin confirmed that in these patients apixaban was not associated with an increase in major bleeding compared to aspirin (4.7%/year on apixaban and 4.9%/year on aspirin) [32]. The ROCKET AF trial demonstrated non-inferiority for rivaroxaban vs. warfarin regarding efficacy and safety in the elderly [33]. The ENGAGE AF trial showed a similar occurrence of stroke with reduced bleeding risk in the elderly with edoxaban versus warfarin [34]. A long-term follow up after patients receiving edoxaban revealed a minimal amount of events of ischemic strokes and major bleedings [35]. A post hoc analysis of the RE-LY study showed that in patients over 75 years, extracranial major bleeding was similar between dabigatran 110 mg BID and warfarin but was more common in patients receiving dabigatran 150 mg BID than warfarin. Intracranial hemorrhage (ICH) risk was lower in the dabigatran group at both doses compared to warfarin [36]. A subsequent retrospective observational analysis of patients older than 80 years old, which compared DOACs vs. warfarin for AF, showed that apixaban had a lower risk of cerebral vascular accident (CVA) or major bleeding, dabigatran had a lower risk of CVA with similar bleeding risk and rivaroxaban was associated with lower risk of CVA but higher risk of major bleeding [37]. The ELDERCARE-AF trial showed that in elderly patients (age > 80), low-dose edoxaban was superior to placebo in preventing stroke or systemic embolism without a significant higher risk of major bleeding [38]. Regarding very elderly patients (age > 90), current data support treatment with anticoagulation. In a retrospective study among 11,064 very elderly patients with AF, treatment with warfarin significantly reduced the risk of ischemic stroke, with no difference in ICH risk compared with nontreatment. DOACs were associated with reduced bleeding risk, without a difference in ischemic stroke risk [39]. In another retrospective study, DOAC treatment led to a reduction in mortality and systemic embolic events in patients with AF aged >90 years, compared to nontreatment. However, DOAC treatment led to increased hemorrhagic events, although not ICH [40].

Despite their improved safety profile when compared to VKAs, underdosing of DOACs in older patients with AF is prevalent. Real-world data showed that practitioners prescribe reduced doses of apixaban and rivaroxaban 4.4 times more than the respective registry trials for these treatments [41]. While not conforming to clinical trial-based evidence, this practice may have some merit because it has been demonstrated that older individuals have higher plasma levels of apixaban than expected when treated with appropriate doses [42]. The same may apply to the other DOACs too.

Underuse of anticoagulants in older patients is also based on the perceived risk related to multiple comorbidities. Yet studies show this risk to be unfounded. In the ARISTOTLE trial the efficacy and safety profile of apixaban vs. warfarin was similar regardless of the presence or absence of multimorbidity [5], as was also the case in the ENGAGE AF-TIMI 48 trial for edoxaban vs. warfarin [29].

Frailty is a clinical condition, characterized by increased vulnerability to stressors and reduced physiologic reserve. Frail patients carry a high risk of stroke and major bleeding [40,43]. In AF patients, frailty is associated with increased risks of ischemic stroke, bleeding and death [43]. In a retrospective cohort, treatment with OAC in frail patients with AF reduced the composite of death, strike, systemic embolism, or major bleeding [44]. In a registry-based cohort of frail patients with AF, treatment with DOACs in regular and reduced doses resulted in comparable thromboembolism risk with lower bleeding risk compared to warfarin [45].

Polypharmacy, a phenomenon common in older patients, increases the risk of major bleeding in patients receiving a DOAC. This is primarily due to concomitant use of other medications and drug to drug interactions, necessitating an individualized approach when prescribing a DOAC in this population [46,47,48]. When the significance of polypharmacy was studied in elderly patients in the ARISTOTLE trial, there was no effect on the risk of stroke or mortality with apixaban vs. warfarin, while the reduction in major bleeding with apixaban was no longer significant in those with nine or more comedications [34]. Nevertheless, in a large retrospective study, polypharmacy was associated with increased risk of thromboembolic events, bleeding and mortality in AF patients. DOACs were found to be associated with lower stroke, systemic thromboembolism, mortality, major bleeding and ICH compared to warfarin, although the GI bleeding risk was higher [49].

It should be noted that in most elderly patients with AF, including those with frailty, risk of falling, comorbidity or polypharmacy, the benefit of DOACs outweigh their risk of bleeding [4]. The exceptions to this are severely frail and dependent patients, and those approaching end of life [10].

*AF in this patient is probably of relatively recent onset since she has had multiple encounters with providers in recent years and has had a pacemaker for a year. The main challenges in this patient are related to her age, frailty, risk of falls, renal function and the possibility of cognitive deterioration in the near future. She would not have been eligible for inclusion in the major RCTs studying DOACs in AF, thus in her case decisions are necessarily based on extrapolation and clinical judgment. She uses a walker and has a higher risk of falls; however, based on clinical data and European guidelines [5], the advantage of anticoagulation in preventing stroke outweighs the risk of major hemorrhage associated with falling. Thus, this patient should receive anticoagulation. When applying the available data DOACs may be a good option for this patient with a better safety profile than warfarin after assessing modifiable bleeding risk factors such as clopidogrel discontinuation and adjusted dose DOAC.*

## 4. Direct Oral Anticoagulants in Patients with Significant Drug–Drug Interactions (DDIs)

DDIs can affect significantly DOAC safety and efficacy [50]. Coadministration of DOACs with antiplatelets medications is known to be associated with increased risk of bleeding [51], and thus should be limited to patients with strong indications for antiplatelets.

Since specific DOACs differ in their metabolic pathways, DOACs’ susceptibility to PK DDIs vary considerably according to their elimination pathways. For example, rivaroxaban and apixaban undergo cytochrome P-450 (CYP450)–mediated metabolism and thus are subject to DDI by strong inhibitors which increase anticoagulant exposure following co-treatment with CYP450 inhibitors (including moderate inhibitors such as dronedarone, verapamil, diltiazem, fluconazole, cyclosporine, amiodarone and ciprofloxacin, and strong inhibitors such as ritonavir, ketoconazole and clarithromycin), resulting in increased bleeding risk [52], while co-treatment with CYP450 inducers (such as phenytoin, phenobarbital, primidone, carbamazepine, oxcarbazepine and St. John’s wort) may result in reduced rivaroxaban and apixaban concentration and increased risk for treatment failure [53]. Dabigatran concentration, which is highly dependent on P-gp mediated absorption, is affected by inhibitors and inducers of this transporter.

## 5. Direct Oral Anticoagulants in Extremes of Weight

*A 64-year-old man weighing 125 kg who is scheduled for bariatric surgery (BS) presents to the emergency department with a right-leg proximal deep vein thrombosis. He has hypertension, obstructive sleep apnea and pulmonary hypertension. His medications include a glucagon-like peptide 1 agonist, an angiotensin converting enzyme inhibitor and a beta-blocker. Laboratory tests show creatinine 1.0 mg/dL, hemoglobin 17 g/dL, LDL 170 mg/dL and fasting glucose 125 mg/dL. In patients with extremely high or low weight, does the use of DOACs compared to alternative OACs demonstrate comparable efficacy and safety?*

DOACs have a rapid onset of action, a broad therapeutic window and minimal interactions with food and other drugs resulting in predictable pharmacokinetics [54]. As a result, they can be prescribed in fixed-dose regimens without the need for routine laboratory monitoring, distinguishing them from VKAs. This advantage stands as one of the significant strengths of DOACs.

However, recent concerns have emerged regarding the safety and efficacy of fixed-dose DOACs in patients at the extremes of weight, particularly in light of the escalating prevalence of obesity [55]. In the United States, where one in three adults receiving medical care is obese [56], it becomes crucial to obtain precise clinical data pertaining to treatment in this population.

Clinicians may exhibit reluctance to initiate DOACs in these patients due to a perception that the fixed dose may not be sufficiently effective for individuals with high body weight or could potentially heighten the bleeding risk in individuals with low body weight. This dilemma has been addressed in laboratory studies, retrospective cohort studies and randomized controlled trials.

Two laboratory studies that investigated the impact of extreme body weights on the pharmacokinetics, pharmacodynamics, safety and tolerability of apixaban and rivaroxaban in healthy individuals [57,58] concluded that no significant changes were observed in plasma concentration, area under the curve, or other clinically relevant factors at the extremes of body weight. Therefore, it was deduced that DOACs are unlikely to require dose adjustment based on body weight. In the study published by Upreti et al. [58], 54 healthy subjects were enrolled and divided into three groups based on body weight (<50 kg, 65–80 kg and >120 kg). Each received a single dose of apixaban 10 mg. The plasma anti-factor Xa activity showed a linear relationship with apixaban plasma concentrations, irrespective of body weight groups, and was in the therapeutic range in all study groups. It was concluded that no dose adjustment is recommended for apixaban based on body weight alone and consequently current guidelines recommend measuring trough plasma levels only in selected patients (Table 4) [12,13].

In 2017, a comprehensive systematic review and meta-analysis [62] encompassing 11 phase III RCTs examined the relationship between body weight and clinical outcomes in over 90,000 patients treated with DOACs or warfarin. The findings indicated an increase in thrombotic risk in low body-weight patients compared to non-low body-weight patients, while there was no risk difference in high body-weight patients compared to non-high body-weight patients. However, no significant differences in the risks of thrombosis and bleeding were attributed to the choice of anticoagulant but rather to the baseline risk in each weight group. Thus, the study concluded that dose adjustment of DOACs outside that recommended in the package insert is unlikely to improve safety or efficacy.

Guidelines caution against the use of DOACs in patients with extremely high (>120 kg) or low (≤60 kg) body weight due to limited available data for these populations. However, a post hoc analysis of the ARISTOTLE trial (with 10.9% weighing <60 kg and 5.4% weighing >120 kg) provided insights into the treatment effect of apixaban compared to warfarin in these populations. The efficacy outcomes of stroke or systemic embolism prevention, all-cause mortality or myocardial infarction were consistent across the weight spectrum, with no significant interaction observed (*p* value > 0.05). Moreover, regarding major bleeding, apixaban was safer than warfarin in all weight categories [63].

Kushnir et al. [64] studied anticoagulant use in 795 morbidly obese patients (Body Mass Index (BMI) ≥ 40 kg/m^2^) who were prescribed rivaroxaban, apixaban or warfarin for the treatment of acute venous thromboembolism (VTE) or stroke prevention in AF. The incidence of recurrent VTE or stroke was similar in all patient groups as was the rate of major bleeding. Notably, among the 429 patients receiving anticoagulants for AF, major bleeding occurred in 3 out of 103 patients on apixaban (2.9%, 95% CI 0.0–6.2), 5 out of 174 on rivaroxaban (2.9%, 0.4–5.4), and 12 out of 152 on warfarin (7.9%, 3.6–12.2), with a *p*-value of 0.063. While not statistically significant, there was nearly a two-fold increase in bleeding with warfarin suggesting DOACs as a convenient and safe alternative. If this observational study is validated by prospective studies, it could potentially provide an opportunity for patients with an extremely high BMI to benefit from more convenient and potentially safer anticoagulants.

Another retrospective cohort study [65] focused on the opposite end of the body weight spectrum—AF patients with a body weight of ≤60 kg who were treated with oral anticoagulants. The findings in this group demonstrated that DOACs had superior effectiveness and safety compared to warfarin with a lower risk of ischemic stroke (HR 0.59, 95% CI: 0.51 to 0.68) and major bleeding (HR 0.70, 95% CI: 0.60 to 0.82).

As noted, the prevalence of obesity continues to rise worldwide [55] with a concomitant increase in the performance of BS, with over 200,000 of these procedures being performed annually in the United States [66]. Obesity and abdominal surgery are both established risk factors for thrombotic complications making patients undergoing BS at particularly high risk for VTE.

Physiologic and anatomic changes following BS may affect gastrointestinal absorption of drugs by several mechanisms [67]; therefore, whether DOACs reach adequate blood levels following BS may be of concern.

Data regarding the use of DOACs in patients with a history of BS remains scarce. The 2021 International Society on Thrombosis and Haemostasias guidelines [68] specifically address DOAC use following BS for treatment/prevention of VTE and recommend treatment with a parenteral anticoagulant in the acute setting with switching to VKA or DOACs to be considered after 4 weeks.

A case control study of anticoagulant blood levels performed by Rottenstreich and colleagues [69] examined 18 post-BS patients and 18 controls matched for age, gender, BMI and renal function. In all patients, DOAC levels were measured at their predicted time of peak concentration. Peak apixaban levels following BS were within the expected range. This could be partly explained by absorption throughout the gastrointestinal tract, mostly within the distal small bowel and some in the proximal colon. Levels of rivaroxaban were significantly lower among the post-BS group, and most of them had levels below the expected range, concordant with the recommendation not to use rivaroxaban post BS [13,70].

This vignette demonstrates not only the challenge in body weight extremes but also the dilemma for anticoagulation following BS. As the efficacy and safety of DOACs is at least comparable with that of VKA across weight categories, this patient may be switched to a DOAC in the stable post-acute phase after BS. However, it is important to consider the type of BS when choosing the specific DOAC.

## 6. DOACs in Recurrent Stroke

*A 64-year-old woman with known paroxysmal AF observed during ambulatory monitoring presents with a recurrent ischemic stroke, having had a CVA two years earlier with residual right hemiparesis. She has hypertension and dyslipidemia. Her medications include a DOAC, a calcium channel blocker and a statin. Laboratory tests show creatinine 1.2 mg/dL, hemoglobin 13 g/dL, and platelets 160,000/μL. In the described patient, are DOACs more effective and safer than other oral anticoagulation alternatives?*

Stroke prevention is the primary goal of anticoagulation in AF which is one of the leading causes of cardioembolic stroke worldwide [1,5]. The greater the total AF burden (i.e., time spent in AF) in a patient, the higher the thromboembolic risk, independent of known stroke risk factors [71]. Guidelines for primary and secondary prevention of ischemic stroke in patients with AF all recommend the use of oral anticoagulation when possible, with preference for DOACs over VKAs in appropriate patients who do not have a mechanical valve or moderate to severe mitral stenosis [5,8,72].

Strategies for secondary prevention of stroke in patients already receiving anticoagulation are not well defined. It is unclear what factors contribute to the occurrence of a stroke under supposedly adequate anticoagulation, whether these patients are at increased risk for additional recurrent events and how secondary prevention should be managed thereafter. One observational study found that the factors contributing to the risk of cardioembolic stroke in patients who are already receiving DOAC therapy were low dose administration, atrial enlargement, hyperlipidemia and high CHA_2_DS_2_-VASc score [73]. It was recently shown that the most common cause of stroke in patients with AF is cardio embolism even when receiving oral anticoagulation therapy [74]. Data from the Swiss stroke registry found that in recurrent stroke prior DOAC therapy, compared to VKA or no anticoagulation, was associated with lower stroke severity, lower rates of thrombolysis, lower large vessel occlusion rates and better functional outcome [75]. The same observation was made in patients with ICH where prior use of DOACs was associated with lower risk of in hospital mortality compared to warfarin [76,77]. A large pooled analysis of seven prospective studies demonstrated that patients who had undergone an ischemic stroke despite anticoagulation were at increased risk for recurrent stroke compared to patients with the same CHA_2_DS_2_-VASc score who were not receiving anticoagulation. Anticoagulation was associated with a lower stroke-severity score on admission, and pretreated patients were less likely to receive thrombolytic therapy. Switching to a different drug did not lower the risk of future ischemic stroke [78].

There is no treatment of choice after ischemic stroke in patients with AF receiving DOAC. In a large retrospective study in this population, switching to warfarin or a different DOAC after the stroke resulted in a higher incidence of ischemic stroke during a 6-year follow up. The addition of antiplatelet did not differ [79]. Thus, there is no clear evidence supporting switching anticoagulants after stroke in patients receiving a DOAC.

The timing of (re-) initiation of anticoagulation after ischemic stroke may affect the prognosis, as anticoagulation reduces the risk of recurrent stroke but increases the bleeding risk in the acute phase in part because of cerebral infarction-induced disruption of the blood–brain barrier [80]. Clear guidance for decision making in this situation is lacking. Factors influencing the decision when to restart anticoagulation are the size of the infarct, with a National Institute of Health Stroke Scale score greater than 15 and infarcted territories involving more than one artery being the operational definitions for a large stroke [13]. A recent clinical trial, comparing early versus later reinitiation of DOACs after ischemic stroke in patients with AF, showed no difference in the incidence of recurrent ischemic stroke, systemic embolism, major extracranial bleeding, symptomatic intracranial hemorrhage, or vascular death at 30 days [81]. A prospective observational study, comparing early (<4 days) versus late (≥4 days) anticoagulation treatment after ischemic stroke, showed results of a greater number of ischemic lesions on MRI after 1 month in the late treatment group, without a difference in bleeding rates [82]. Early treatment with edoxaban after ischemic stroke demonstrated good safety features in patients after cardioembolic stroke [83]. Current acceptable practice guidelines state that in most patients (re-) initiation of anticoagulation is reasonable between 4 and 14 days of the stroke, with longer delays after larger infarcts [8,13,84].

In patients with a contraindication to lifelong anticoagulation, source-specific treatment may be implemented. Over 90% of ischemic strokes of cardioembolic origin stem from a formed thrombus in the left atrial appendage (LAA) [85], making LAA closure an alternative strategy for stroke prevention. Two studies, PREVAIL and PROTECT AF, demonstrated comparable stroke prevention using LAA closure compared to warfarin with reduced bleeding complications [86]. In the PRAGUE-17 trial, 402 patients with high ischemic risk and high bleeding risk were randomized to receive either DOAC or percutaneous LAA closure. LAA closure was non-inferior to DOAC in preventing AF-related cardiovascular and neurological embolic events and was associated with statistically nonsignificant less bleeding. However, safety concerns regarding procedural complications and device-related thrombosis necessitating short-term anticoagulation and long-term antiplatelet therapy have been obstacles to this procedure’s widespread acceptance [5,9,87].

*This patient’s clinical presentation poses a great clinical challenge in the decision of timing of reinitiating anticoagulation and the choice of treatment. Given that the risk for future ischemic events is high and based on European guidelines* [5]*, adherence to medical treatment and specifically her DOAC should be emphasized, and optimal dosing and treatment intervals should be assessed. Reinitiating her current medication following a timely delay may be appropriate.*

## 7. DOACs after Major Hemorrhage

*A 74-year-old man presents to the emergency department with acute-onset, severe headache. His medical history is significant for permanent AF, hypertension, dyslipidemia, mild to moderate mitral stenosis, iron deficiency anemia and ischemic heart disease with a right coronary artery stent. His medications include warfarin, doxazosin, atorvastatin, and metoprolol. Laboratory tests show creatinine 0.88 mg/dL, hemoglobin 11 g/dL and INR 3.4. Head computerized tomography (CT) shows a small cerebral hemorrhage in the parietal lobe. He receives prothrombin complex concentrate with no expansion of the bleeding on a repeat CT scan. For patients with major intra or extracranial hemorrhage, what is the preferred option as anticoagulation if appropriate?*

The use of DOACs in patients without previous ICH is associated with an approximately 50% lower risk of ICH compared to VKAs [88]. Despite their favorable safety profile, ICH occurs at a rate of approximately 0.5% per year [89]. In patients with AF with a high risk of ischemic stroke in whom ICH has occurred previously, the ESC guidelines recommend DOACs over VKA based on their safety profile in patients without a history of ICH and no RCT has proven this choice [5]. However, prescribing anticoagulation in this setting is a significant concern, especially because patients with a history of recent ICH were excluded from most RCTs of stroke prevention in AF.

In a large population-based cohort study that examined 77,834 head injury cases among older patients, the relative risk for ICH was similar between patients not on an anticoagulant and patients on DOACs. In a cohort of 219,701 patients with nontraumatic ICH, patients on factor Xa inhibitors (rivaroxaban, apixaban or edoxaban) had a significantly lower mortality risk than patients on warfarin (adjusted OR 0.76; 95% CI, 0.72–0.81; *p* < 0.001) [89]. In cases of active bleeding, DOACs must be withheld and in severe bleeding anticoagulant-reversal agents should be administered. Idarucizumab was developed as a specific antidote to dabigatran, reversing the anticoagulant effectively within minutes [90]. Reestablishment of hemostasis in patients with ICH while on factor Xa inhibitors may be achieved using prothrombin complex concentrate or the specific reversal agent, andexanet alfa, though the latter is not universally approved [91].

The more complex decision is whether and when to recommence anticoagulation treatment after the acute phase, and conflicting data regarding this issue have been published [92]. Data from a large Swedish registry suggest that the greatest benefit in anticoagulation in this setting is achieved when treatment is restarted 7 to 8 weeks after ICH [93]. On the other hand, the phase 2 APACHE-AF trial found no difference in the annual risk of non-fatal stroke or vascular death in patients after ICH whether on apixaban or on no anticoagulation [94]. As data are still lacking, the ESC guidelines recommend anticoagulation resumption after the acute phase of the ICH at least 4 weeks after the event. LAA occlusion should be considered in patients with a particularly high risk of ICH recurrence [5].

While DOACs are associated with a lower incidence of ICH than VKAs, there is conflicting evidence relating to their safety regarding gastrointestinal bleeding (GIB). Observational studies suggest increased GIB with their use [88] while a subgroup analysis of the ARISTOTLE trial showed that patients with prior GIB had a higher risk of major GIB after initiating anticoagulation, with no difference in bleeding rates between apixaban and warfarin. Prior upper GIB but not lower GIB was associated with higher rates of major GIB after anticoagulation initiation [95]. In a large Korean claims database, patients with previous GIB had a lower risk of recurrence with the initiation of DOACs compared to warfarin [96].

While no head-to-head direct comparisons in rates of GIB between DOACs are available, a network meta-analysis showed that rivaroxaban was associated with higher rates of major GIB compared to other DOACs. Apixaban and edoxaban showed a significantly lower risk of massive GIB, and apixaban had the highest probability of being the safest option in the context of major GIB [97]. These results were recently supported in a large multinational population-based cohort study where apixaban use was associated with lower GIB risk and similar rates of ischemic stroke or systemic embolism, ICH and all-cause mortality compared to dabigatran, edoxaban and rivaroxaban [98].

Importantly, although continuation of DOAC therapy after GIB is a risk factor for recurrence of bleeding, it is also associated with a reduction in mortality [99]. Thus, each patient should be assessed according to the severity of the bleeding event and the underlying thromboembolic risk. While optimal timing of reinitiating anticoagulation is unclear, the available evidence regarding anticoagulation resumption after a life-threatening bleeding event favors resumption as a default strategy. After weighing the thromboembolic risk and underlying comorbid risk factors, most studies indicate that re-starting anticoagulation two weeks after major GIB and one month after ICH may optimize clinical outcomes [100].

*In this patient the anticoagulant effect of warfarin was appropriately reversed using a four-factor PCC. Her high CHA_2_DS_2_-VASc score places her at high risk for another stroke, and therefore continued anticoagulation using a DOAC rather than a VKA, beginning at least* 4 *weeks after the current ICH may be appropriate, based on European (class* 2a *recommendation)* [5] *and American (class* 2b *recommendation)* [26] *guidelines. LAA occlusion may be considered as an alternative to long-term anticoagulation.*

## 8. DOACs in Patients with Cancer

*A 62-year-old man presents to the clinic with palpitations. He was diagnosed 5 months prior to his presentation with non-small cell lung cancer and treatment with cisplatin and gemcitabine was initiated. His past medical history is significant for hypertension and gout. His medications include valsartan and allopurinol. ECG shows atrial fibrillation. In patients with AF and cancer, is the use of DOACs superior to other treatment options in preventing thromboembolic and bleeding complications?*

Atrial fibrillation poses a unique challenge in cancer patients. It may appear initially in correlation with the cancer or one of its medical therapies. Some anticancer medications are associated with an increased risk of AF [101]. In addition, anticoagulation management is complex, as the thromboembolic risk is high in these patients and the CHA_2_DS_2_-VASc score is not validated for cancer patients and usually underestimates the thromboembolic risk [102].

DOACs have been studied in oncologic patients. However, data from RCTs are only available in the treatment of VTE. Apixaban was found to be noninferior to dalteparin in preventing VTE recurrence (*p* < 0.001 for noninferiority), without an increased risk of major bleeding [103]. In another trial comparing apixaban to dalteparin, there was 0% of major bleeding events in the apixaban group over 6 months, compared to 1.4% in the dalteparin group. No significant difference was found in VTE recurrence [104]. Another study showed the superiority of rivaroxaban over dalteparin in preventing VTE recurrence (HR 0.43; 95% CI, 0.19 to 0.99) without a significant difference in major bleeding [105]. Edoxaban was also studied in comparison to dalteparin for the prevention of VTE recurrence, demonstrating noninferiority (*p* = 0.006) without an increase in bleeding risk [106].

Data regarding prevention of systemic thromboembolism due to AF in patients with cancer are lacking. To date, no RCTs dealing with this question have been published. A large metanalysis of over 220,000 patients with non-valvular AF and cancer demonstrated that DOACs were associated with a significant reduction in the rates of thromboembolic events and major bleeding complications compared to VKAs [107].

*This patient’s CHA_2_DS_2_-VASc score is 1 (hypertension), and according to the European guidelines [101], anticoagulation should be considered. Treatment options include VKA, DOAC, LMWH or an LAA occluder.*

## 9. Summary

One of the cornerstones of treatment in patients with AF is anticoagulation for stroke prevention. During the past decade, DOACs have largely replaced VKAs as the anticoagulant of choice based on the results of pivotal randomized clinical trials. However, in these trials, some high-risk populations have been systematically underrepresented, despite the higher prevalence of AF in these groups. In patients with advanced renal impairment, where the AF burden is greater than in the general population, the current use of DOACs is limited due to a knowledge gap regarding the safety and efficacy in these patients. Nonetheless data have accrued suggesting that apixaban has a better efficacy and safety profile than other anticoagulants in this setting. In post hoc analyses of older patients treated in all four pivotal trials comparing DOACs to VKAs, outcomes of stroke and major bleeding were comparable, with some advantages shown in the apixaban groups. Lack of sufficient data regarding the use of DOACs in extremes of body weight have limited their use in patients weighing less than 60 kg or more than 120 kg. However, post hoc analyses and retrospective data have demonstrated comparable safety and efficacy to VKAs in all weight ranges. The challenge of secondary prevention in patients presenting with recurrent stroke, especially in patients already receiving anticoagulation, remains significant. Dose reduction of DOACs and higher CHA_2_DS_2_-VASc are important risk factors for stroke in patients already receiving anticoagulation. In these patients, DOACs are associated with a lower stroke severity and better outcomes compared to VKAs. LAA occlusion devices may be a feasible alternative in patients who cannot tolerate or who fail anticoagulation; however, data regarding the safety of this procedure are conflicting. The more favorable safety profile for DOACs has encouraged current guidelines to recommend DOACs over VKAs in patients who have experienced an ICH. Data for major GIB, however, are not as compelling, as some observations have shown a greater risk in patients using DOACs. However, some data suggest a lower GIB risk in patients using edoxaban and apixaban. Taken together, these data may allow clinicians to extend the use of DOACs to these high-risk patient populations.

## Figures and Tables

**Table 1:** Common DOACs with recommended dose adjustments. Cr, creatinine; CrCl, creatinine clearance.

Apixaban		Dabigatran		Edoxaban		Rivaroxaban	
Criteria	Dose	CrCl (mL/min)	Dose	CrCl (mL/min)	Dose	CrCl (mL/min)	Dose
Regular	5 mg BID	>30	150 mg BID	>95	NA	>50	20 mg QD
		>30 (high bleeding risk)	110 mg BID				
2/3:Age >80Cr > 1.5 mg/dL weight < 60 kg	2.5 mg BID	15–30	75 mg BID	50–95	60 mg QD	15–50	15 mg QD
Hemodialysisand: Age < 80 or weight > 60 kg	5 mg BID	<15	NA	15–50	30 mg QD	<15	NA

**Table 2:** Major studies of DOACs in patients with chronic kidney disease. eGFR, estimated glomerular filtration rate; RCT, randomized controlled trial.

Study	Participants	Study Type	DOAC	Comparator	Inclusion Criteria	Summary
Yao X (2020) [20]	34,569 patients divided by eGFR	Retrospective	apixaban, dabigatran, rivaroxaban	warfarin	eGFR ≥ 15 mL/min/1.73 m^2^	DOACs were associated with comparative or better outcomes across all ranges of kidney function
Pokorney SD (2022) [21]	154 hemodialysis patients	RCT	apixaban	warfarin	Hemodialysis	Inadequate power to draw conclusion
Reinecke H (2023) [22]	97 hemodialysis patients	RCT	apixaban	phenprocoumon	Hemodialysis	No difference in safety or efficacy
Kuno T (2020) [24]	71,877 hemodialysis patients	Meta-analysis	apixaban, dabigatran, rivaroxaban	warfarin	Hemodialysis	No difference in efficacy, apixaban had lower bleeding risk
Siontis KC (2018) [25]	25,523 hemodialysis patients	Retrospective	apixaban	warfarin	Hemodialysis	No difference in stroke/thromboembolic events, apixaban had lower bleeding risk. Apixaban 5 mg bid showed lower rates of stroke/thromboembolic events/death with no difference in bleeding

**Table 3:** Randomized controlled trials of DOACs in advanced age. ICH, intracranial hemorrhage; OAC, oral anticoagulant; RCT, randomized controlled trial; VKA, vitamin k antagonist.

Study	Participants	Study Type	DOAC	Comparator	Inclusion Criteria	Summary
Ng KH (2016) [32] [AVERROES]	1898 patients age > 75 366 patients age > 85	RCT	apixaban	aspirin	Age > 75 and unsuitable for VKA treatment	Apixaban treatment resulted in lower stroke rates without difference in major hemorrhage rates compared to aspirin
Halperin JL (2014) [33] [ROCKET AF]	6229 patients age > 75	RCT	rivaroxaban	warfarin	Age > 75	Elderly patients had higher rates of stroke and major hemorrhage with no difference between rivaroxaban and warfarin
Kato ET el al. (2016) [34] [ENGAGE AF-TIMI 48]	8474 patients age > 75	RCT	edoxaban	warfarin	Age > 75	Same rates of stroke/systemic embolism with lower bleeding rate in the edoxaban group
Eikelboom JW et al. (2011) [36] [RE-LY]	7248 patients age > 75	RCT	dabigatran (150 mg, 110 mg)	warfarin	Age > 75	Both doses of dabigatran were associated with lower ICHand higher extra-cranial hemorrhage compared to warfarin
Okumura K et al. (2020) [38]	984 patients age > 80	RCT	low-dose edoxaban	placebo	Age > 80 and unsuitable for OAC in standard dose	Low-dose edoxaban was superior to placebo in preventing stroke or systemic embolism without a significant elevation in major bleeding

**Table 4:** Pharmacokinetic and pharmacodynamic trials of DOACs with weight considerations. AUC, area under the curve; PT, prothrombin time.

Study	Participants	DOAC	Summary
Barsam J et al. (2017) [59]	101 patients	rivaroxaban	Weight had no effect on pharmacokinetics and pharmacodynamics
Kubitza D et al. (2007) [57]	48 healthy subjects divided to groups by weight: <50 kg, 70–80 kg, >120 kg	rivaroxaban	Cmax of rivaroxaban was unaffected in subjects > 120 kg but was increased by 24% in subjects < 50 kg resulting in 15% increase in PT. AUC was unaffected by weight
Liesenfeld KH (2011) [60]	9522 patients	dabigatran	Weight influenced the apparent volume of distribution with no effect on the AUC
Upreti VV et al. (2013) [58]	54 healthy subjects divided to groups by weight:<50 kg, 65–85 kg, >120 kg	apixaban	Low body-weight group had 27% higher Cmax and 20% higher AUC (0, ∞). High body-weight group had 31% lower Cmax and 23% lower AUC (0, ∞).
Yamashita et al. (2012) [61]	536 patients	edoxaban	Cmin was higher in patients < 60 kg

## Abbreviations

AF; atrial fibrillation, CHA_2_DS_2_-VASc; congestive heart failure, hypertension, age, diabetes mellitus, prior stroke or transient ischemic attack, vascular disease and sex category, DOACs; direct oral anticoagulants, BID; twice daily, RCT; randomized controlled trial, VKA; vitamin K antagonist, ICH; intracranial hemorrhage, BS; bariatric surgery, VTE; venous thromboembolism, GIB; gastrointestinal bleeding.
